# Telemedicine for cognitive impairment: a telephone survey of patients’ experiences with neurological video consultation

**DOI:** 10.1007/s10072-023-06903-9

**Published:** 2023-06-27

**Authors:** Fabiana Ruggiero, Eleonora Zirone, Maria Takeko Molisso, Tiziana Carandini, Giorgio Fumagalli, Anna Pietroboni, Roberta Ferrucci, Edoardo Nicolò Aiello, Barbara Poletti, Vincenzo Silani, Giacomo Comi, Elio Scarpini, Sergio Barbieri, Andrea Arighi, Francesca Mameli

**Affiliations:** 1 Department of Neuroscience and Mental Health, Foundation IRCCS Ca’ Granda Ospedale Maggiore Policlinico, Milan, Italy; 2 Department of Oncology and Hemato-Oncology, University of Milan, Milan, Italy; 3 Department of Neurology and Laboratory of Neuroscience, IRCCS Istituto Auxologico Italiano, Milan, Italy; 4 Department of Pathophysiology and Transplantation (DEPT), Dino Ferrari Centre, University of Milan, Neuroscience Section, Milan, Italy; 5 ASST Santi Paolo e Carlo, Milan, Italy

**Keywords:** Cognitive impairment, Dementia, Caregiver, COVID-19, Telemedicine, Neurological video consultation

## Abstract

**Objective:**

This study aimed to evaluate the experience with telemedicine in patients with cognitive impairments and their caregivers.

**Methods:**

We conducted a survey-based study of patients who completed neurological consultation via video link between January and April 2022.

**Results:**

A total of 62 eligible neurological video consultations were conducted for the following categories of patients: Alzheimer’s disease (33.87%), amnesic mild cognitive impairment (24.19%), frontotemporal dementia (17.74%), Lewy body dementia (4.84%), mixed dementia (3.23%), subjective memory disorders (12.90%), non-amnesic mild cognitive impairment (1.61%), and multiple system atrophy (1.61%).

The survey was successfully completed by 87.10% of the caregivers and directly by the patients in 12.90% of cases. Our data showed positive feedback regarding the telemedicine experience; both caregivers and patients reported that they found neurological video consultation useful (caregivers: 87.04%, ‘very useful’; patients: 87.50%, ‘very useful’) and were satisfied overall (caregivers: 90.74%, ‘very satisfied’; patients: 100%, ‘very satisfied’). Finally, all caregivers (100%) agreed that neurological video consultation was a useful tool to reduce their burden (Visual Analogue Scale mean ± SD: 8.56 ± 0.69).

**Conclusions:**

Telemedicine is well received by patients and their caregivers. However, successful delivery incorporates support from staff and care partners to navigate technologies. The exclusion of older adults with cognitive impairment in developing telemedicine systems may further exacerbate access to care in this population. Adapting technologies to the needs of patients and their caregivers is critical for the advancement of accessible dementia care through telemedicine.

## Introduction

The coronavirus disease (COVID-19) pandemic has rendered older adults more vulnerable to not receiving the healthcare needed. Furthermore, it has placed those living with dementia at an even increased risk of developing other mental health symptoms due to social isolation and loneliness [1]. Indeed, as the virus spreads, it has become necessary to introduce social distancing measures, such as quarantine within urban areas, prohibition of travel to and from certain countries, and suspension of a large range of clinical activities. Elective face-to-face consultations had to be rescheduled, and the need for health care during the pandemic required telehealth solutions. National initiatives have been launched to review and update previous restrictions on telehealth practices and to expand their use as a solution for improving the care provided in the national state-funded health system [2].

Older adults were considered to be the most vulnerable portion of the population at the onset of the COVID-19 pandemic. This situation has raised concerns regarding the management and care of people with cognitive impairment and their caregivers. Behavioural and psychological symptoms of dementia can be triggered or exacerbated by several risk factors, such as social isolation, pharmacology adherence disruption, caregiver load, reduction of non-pharmacologic techniques, absence of medical assessment, and changes in home routine [2].

Telehealth has proven to be a viable alternative for providing individuals with appropriate services and care, as well as for mitigating the effects of social isolation, particularly in older patients with cognitive impairment and dementia [3, 4]. Through video consultation systems, telemedicine allows healthcare professionals to manage patients remotely and conduct follow-up and monitoring visits, enabling patients to receive continuous care support [5].

With the growing population of older adults with dementia, it is imperative to improve telemedicine systems to adapt them to their needs. To the best of our knowledge, few studies have assessed the critical issues of using neurological video consultation to deliver care to patients with cognitive impairment or dementia in the Italian public health system.

This study aimed to evaluate the experience of telemedicine in patients with cognitive deficits and their caregivers to outline new socio-health models to meet the care needs of fragile patients, as in the case of neurodegenerative diseases.

## Methods

### Recruitment

We conducted an institutional review board-approved survey-based study of patients who completed routine neurological consultation via video link between January 2022 and April 2022 at the Alzheimer’s Unit of the IRCCS Ca’ Granda Foundation Ospedale Maggiore Policlinico in Milan, Italy. The patients were being treated for amnesic and non-amnesic mild cognitive impairment (MCI), Alzheimer’s disease (AD), Lewy body dementia (LBD), frontotemporal dementia (FTD), mixed dementia, multiple system atrophy (MSA), and subjective memory disorders. The survey was administered to the caregivers of all patients diagnosed with cognitive impairment, whereas the survey was proposed directly to those patients diagnosed with subjective memory disorders who attended video consultation without the support of a caregiver.

The extent to which the caregivers’ experienced a reduction in burden after the neurological video consultation was assessed using a Visual Analogue Scale (VAS) with items rated from 0 (not at all) to 10 (complete reduction in burden).

The participants were contacted by telephone using their preferred contact phone number provided in the electronic medical record, by two psychologists within 1 week of their neurological video consultation. The interviewers were responsible for conducting the pilot test calls, making final phone calls, and recording data for analysis. The database containing the total sample was organised alphabetically according to diagnosis and divided into halves, each of which was distributed to the interviewers for the telephone survey.

The appropriate Ethics Committee reviewed and approved the distribution of the survey for this study following local legal standards and authorised the disclosure (Protocol no. 184_2021bis). Due to the COVID-19 pandemic, consent for the processing of personal data was obtained from the participants over the phone before the survey.

### Survey development and structure

We created a survey based on the recommendations proposed by Langbecker et al. [6], who investigated the most common surveys and tools in remote medical research.

The survey consisted of two sections, as described in our previous study [7]. The first section was used to collect clinical and demographic data. The second section evaluated participants’ viewpoint to video consultation (defined as ‘telemedicine’) through the following constructs: *‘satisfaction’, ‘experience’, ‘technical quality’, ‘effectiveness’, ‘usefulness/perceived usefulness’,* and *‘effect on interaction’* [6] (Table 1). Participants were asked to rate their agreement with multiple statements on a VAS ranging from 0 (‘strongly disagree’) to 4 (‘strongly agree’).

**Table 1:** Survey constructs

	Definition	Indicators variable
Satisfaction	Subjective evaluation of whether the user’s expectations were met	Overall satisfaction Willingness to use/re-use
Experience	Evaluation of the user’s experience of a healthcare service. The experience can be objective (e.g., waiting time) or subjective (e.g., level of patient-centeredness)	Patient-reported experience measures Comfort Patient-centeredness
Technical Quality	Subjective evaluation of the quality of the technology used	Audio and picture (Video) Quality Reliability Usability/Ease of use Intuitiveness/Learnability Privacy and Security
Effectiveness	Objective/subjective assessment that a telehealth interaction helped improve the health status or well-being of a patient	Quality of life Change in health status Measures of health/well-being Patient empowerment Patient knowledge Patient-reported outcome measures
Usefulness/ Perceived Usefulness	Objective or subjective assessment that a telehealth interaction produced some benefit or met the purpose of the interaction	Convenience Time consequences Cost consequences Accessibility Effect on continuity of care (Future) intention to use Willingness to use/practice Acceptability
Effect on Interaction	Effect on interaction	Communication style Ease of communication Completeness of information

### Statistical analysis

Responses were recorded using REDCap electronic data capture tools hosted at the Policlinico Hospital which was also used for data entry, editing, and sorting. For all continuous variables, the mean and standard deviation (SD) were calculated, and categorical data were represented as frequencies and percentages. Statistical analysis of the data was performed using TIBCO Statistica ™ software (version 13.5). Wilcoxon Rank Sum test and Chi-Square test were applied to compare, respectively, continuous and categorical variables between groups. To evaluate the correlation between the construct scores of the survey, Spearman’s rank correlation coefficient (r) was calculated between each construct and all others. In this study, we considered both positive and negative correlations with significance ranging from r = 0.5 to r = 0.7. Statistical significance was set at *p* < 0.05.

## Results

During the study period, 83 patients underwent video neurological consultations. Among them, 21 (25.30%) were excluded from the survey-based study: 19 (22.89%) chose not to participate, and 2 (2.41%) were unreachable by phone.

We collected data from 62 respondents (74.70%): 54 (65.10%) were caregivers and 8 (9.64%) were patients. No significant differences we found between the demographic and clinical characteristics of the patients of caregivers and patients who responded independently to the survey with those of the excluded patients (Table 2).

**Table 2:** Demographic and clinical data of study participants and non-participants to survey-based study

Demographic and clinical data		62 participants	21 non-participants	*p value*
Age (years)^a^		73.16 ± 9.05	73.52 ± 5.94	*p* = *0.51*
Gender^b^	Male	31 (50.00)	10 (47.62)	*p* = *1*
	Female	31 (50.00)	11 (52.38)	
MMSE score^a^		18.87 ± 8.57	19.95 ± 7.38	*p* = *0.74*
MMSE score compared to cutoff^b^	< 23.80 ≥ 23.80	38 (61.29) 24 (38.71)	15 (71.43) 6 (28.57)	*p* = *0.57*
Disease diagnosis^b^	Alzheimer’s disease	21 (33.87)	12 (57.14)	*p* = *0.34*
	Amnesic mild cognitive impairment	15 (24.19)	1 (4.76)	
	Frontotemporal dementia	11 (17.74)	4 (19.05)	
	Lewy body dementia	3 (4.84)	0 (0.00)	
	Mixed dementia	2 (3.23)	0 (0.00)	
	Subjective memory disorders	8 (12.90)	4 (19.05)	
	Non amnesic mild cognitive impairment	1 (1.61)	0 (0.00)	
	Multiple system atrophy	1 (1.61)	0 (0.00)	

^a^Data of continuous variables are expressed as mean ± standard deviation and Wilcoxon Rank Sum test is applied to compare the groups

^b^Data of categorical variables are expressed as frequency (%) and Chi-Square test is applied to compare the groups

^c^MMSE: Mini Mental State Examination

To better understand the answers provided by the caregivers of patients with cognitive impairment compared to those of patients with subjective memory disorders, we separately analysed the data of the two groups.

### Caregivers

A total of 54 caregivers (42 women; mean ± SD, age: 56.69 ± 13.98 years; education: 13.91 ± 4.14 years) who were present during the telemedicine session answered the telephone survey. Overall, 26 caregivers (48.15%) belonged to the same generation as the patients or were partners (46.30%) or siblings (1.85%), whereas 28 (51.85%) were part of the younger generation: sons (44.44%), nephews (3.70%), or in-home nurses (3.70%). Twenty-nine caregivers (53.70%) were employed, 21 (38.89%) were retired, and 4 (7.41%) were unemployed.

Most caregivers (87.04%) reported that they did not need technical assistance for setting up the neurological video consultation with our outpatient services, whereas 12.96% of them requested assistance. All caregivers who required technical assistance for telemedicine belonged to the same generation as the patients. Only one participant reported having conducted a neurological video consultation before the COVID-19 pandemic (Table 3).

**Table 3:** (A) Demographic data of caregivers; (B) Demographic and clinical data of patients assisted by caregivers

A. Demographic data of caregivers		54 Caregivers
Age (years)^a^		56.69 ± 13.98
Education (years)^a^		13.91 ± 4.14
Gender^b^	Male	12 (22.22)
	Female	42 (77.78)
Occupation^b^	Working	29 (53.70)
	Retired	21 (38.89)
	Unemployed	4 (7.41)
Family relationship^b^	Partner	25 (46.30)
	Son	24 (44.44)
	Sibling	1 (1.85)
	Nephew	2 (3.70)
	In-home nurse	2 (3.70)
	Other	0 (0.00)
Generation^b,c^	Same generation of patient	26 (48.15)
	Younger generation	28 (51.85)
Previous use of telemedicine^b^	Yes	1 (1.85)
	No	53 (98.15)
Telemedicine technical support^b^	Yes	7 (12.96)
	No	47 (87.04)
Telemedicine technical support: caregivers’ generation^b,c^	Same generation of patient	7 (100.00)
	Younger generation	0 (0.00)
Role of technical assistant^b^	Partner	0 (0.00)
	Son	4 (57.14)
	Sibling	1 (14.28)
	Nephew	1 (14.28)
	In-home nurse	0 (0.00)
	Other	1 (14.28)
Age of technical assistant (years)^a^		40.57 ± 9.95
Education of technical assistant^b^	Primary school license	0 (0.00)
	Secondary school degree	0 (0.00)
	High school degree	2 (28.57)
	College degree	4 (57.14)
	NA^e^	1 (14.28)
B. Demographic and clinical data of patients assisted by caregivers		54 patients
Age (years)^a^		73.19 ± 9.2
Education (years)^a^		10.80 ± 4.54
Gender^b^	Male	27 (50.00)
	Female	27 (50.00)
Occupation^b^	Working	0 (0.00)
	Retired	47 (87.04)
	Unemployed	7 (12.96)
MMSE score^a,d^		17.39 ± 8.19
MMSE score compared to cutoff^b,d^	< 23.80	38 (70.37)
	≥ 23.80	16 (29.63)
Disease diagnosis^b^	Alzheimer’s disease	21 (38.89)
	Amnesic mild cognitive impairment	15 (27.78)
	Frontotemporal dementia	11 (20.37)
	Lewy body dementia	3 (5.56)
	Mixed dementia	2 (3.70)
	Subjective memory disorders	0 (0.00)
	Non amnesic mild cognitive impairment	1 (1.85)
	Multiple system atrophy	1 (1.85)
Disease duration (years)^a^		5.28 ± 2.12
Behavioural disorders perceived by the caregiver^b^	Slight	14 (25.93)
	Moderate	14 (25.93)
	Severe	3 (5.56)
	Absent	23 (42.59)
Area of residency^b^	Northern Italy	54 (100.00)
	Central-Southern Italy	0 (0.00)
Neurological assistance at area of residency^b^	Yes	51 (94.44)
	No	3 (5.56)
Area of neurological assistance^b^	Northern Italy	54 (100.00)
	Central-Southern Italy	0 (0.00)

^a^Data are expressed as mean ± standard deviation

^b^Data are expressed as frequency (%)

^c^Same generation of the patient (partner, sibling), younger generation (son, nephew and in-home nurse)

^d^MMSE: Mini Mental State Examination

^e^NA: data not available

The group of patients (27 women; mean ± SD, age: 73.19 ± 9.2 years; education: 10.80 ± 4.54 years; disease duration: 5.28 ± 2.12 years; Mini Mental State Examination (MMSE) score: 17.39 ± 8.19) for whom the caregivers provided survey responses had a diagnosis of AD (38.89%), amnesic MCI (27.78%), FTD (20.37%), LBD (5.56%), mixed dementia (3.70%), non-amnesic MCI (1.85%), and MSA (1.85%).

In 42.59% of the cases, caregivers reported that the patients did not have behavioural disorders, 25.93% showed slight disturbances, 25.93% showed moderate disturbances, and 5.56% had severe behavioural disorders (Table 3).

According to the responses collected on the *‘usefulness/perceived usefulness’* construct investigated by the telephone survey, caregivers reported that telemedicine was useful (87.04%, ‘very useful’; 9.26%, ‘quite useful’; and 3.70%, ‘neither useful nor useless’). All caregivers reported positive feedback regarding ‘*technical quality*’ (88.89%, ‘very good’ and 11.11%, ‘quite good’). Regarding ‘*effectiveness*’, most caregivers found telemedicine ‘very effective’ (68.52%), 11.11% found it ‘quite effective’, whereas 16.67% did not express a preference and 3.70% found it ‘very ineffective’. Most caregivers also perceived the ‘*effect on interaction*’ with neurologists as ‘excellent’ (88.89%) or of good quality (9.26%), whereas only one reported an interaction of poor quality (1.85%). Most caregivers ranked the ‘*experience*’ positively (79.63%, ‘very good’; 14.81%, ‘quite good’; and 5.56%, did not express a preference). Regarding ‘*satisfaction*’, caregivers were satisfied using telemedicine (90.74%, ‘very satisfied’; 7.41%, ‘quite satisfied’; and 1.85%, ‘neither satisfied nor unsatisfied’) (Table 4).

**Table 4:** Construct response data of caregivers and patients who independently completed the telephone survey

		54 Caregivers	8 Patients	*p value*
		n (%)	n (%)	
Usefulness/Perceived Usefulness	Strongly disagree	0 (0.00)	0 (0.00)	*p* = *1*
	Quite in disagreement	0 (0.00)	0 (0.00)	
	Neither in agreement nor disagreement	2 (3.70)	0 (0.00)	
	Quite in agreement	5 (9.26)	1 (12.50)	
	Strongly agree	47 (87.04)	7 (87.50)	
Technical Quality	Strongly disagree	0 (0.00)	0 (0.00)	*p* = *1*
	Quite in disagreement	0 (0.00)	0 (0.00)	
	Neither in agreement nor disagreement	0 (0.00)	0 (0.00)	
	Quite in agreement	6 (11.11)	1 (12.50)	
	Strongly agree	48 (88.89)	7 (87.50)	
Effectiveness	Strongly disagree	2 (3.70)	0 (0.00)	*p* = *0.60*
	Quite in disagreement	0 (0.00)	0 (0.00)	
	Neither in agreement nor disagreement	9 (16.67)	0 (0.00)	
	Quite in agreement	6 (11.11)	1 (12.50)	
	Strongly agree	37 (68.52)	7 (87.50)	
Effect on Interaction	Strongly disagree	0 (0.00)	0 (0.00)	*p* = *1*
	Quite in disagreement	1 (1.85)	0 (0.00)	
	Neither in agreement nor disagreement	0 (0.00)	0 (0.00)	
	Quite in agreement	5 (9.26)	0 (0.00)	
	Strongly agree	48 (88.89)	8 (100.00)	
Experience	Strongly disagree	0 (0.00)	0 (0.00)	*p* = *1*
	Quite in disagreement	0 (0.00)	0 (0.00)	
	Neither in agreement nor disagreement	3 (5.56)	0 (0.00)	
	Quite in agreement	8 (14.81)	1 (12.50)	
	Strongly agree	43 (79.63)	7 (87.50)	
Satisfaction	Strongly disagree	0 (0.00)	0 (0.00)	*p* = *1*
	Quite in disagreement	0 (0.00)	0 (0.00)	
	Neither in agreement nor disagreement	1 (1.85)	0 (0.00)	
	Quite in agreement	4 (7.41)	0 (0.00)	
	Strongly agree	49 (90.74)	8 (100.00)	

Chi-Square test was used to compare constructs responses between groups

Spearman’s correlation highlighted that ‘*usefulness/perceived* *usefulness’* was moderately correlated with ‘*satisfaction*’ (r = 0.635, p < 0.001). Moderate correlations were found between ‘*effectiveness’* and ‘*experience*’ (r = 0.584, *p* < 0.001), and between the ‘*effect on interaction*’ and ‘*satisfaction*’ constructs (r = 0.514, *p* < 0.001) (Table 5).

**Table 5:** Correlations between construct response data of caregivers

	Usefulness/ Perceived Usefulness	Technical Quality	Effectiveness	Effect on Interaction	Experience
Technical Quality	r = 0.032 *p* = *0.816*				
Effectiveness	r = 0.279 *p* = *0.041*	r = 0.139 *p* = *0.313*			
Effect on Interaction	r = 0.403 *p* = *0.003*	r = 0.243 *p* = *0.077*	r = 0.423 *p* = *0.002*		
Experience	r = 0.127 *p* = *0.359*	r = 0.264 *p* = *0.054*	r = 0.584 *p* < 0.001*	r = 0.303 *p* = *0.026*	
Satisfaction	r = 0.635 *p* < 0.001*	r = 0.087 *p* = *0.533*	r = 0.443 *p* = *0.001*	r = 0.514 *p* < 0.001*	r = 0.212 *p* = *0.124*

^*^Spearman’s rank coefficient r ≥ 0.5 and *p* < 0.05 indicate statistical significance

Finally, all 54 caregivers (100%) agreed that neurological video consultation was a useful tool to reduce their burden (VAS mean ± SD: 8.56 ± 0.69).

### Patients

Only eight patients diagnosed with subjective memory disorders responded independently to the survey (4 women; mean ± SD, age: 73.00 ± 8.52 years; education: 14.25 ± 2.31 years; disease duration: 3.86 ± 0.9 years; MMSE score: 28.88 ± 0.83). Five patients (62.50%) did not report perceiving behavioural disorders; instead, three patients reported the presence of slight-to-moderate behavioural disorders (slight degree in two patients (25.00%) and moderate degree in one patient (12.50%)).

None of these patients reported having used telemedicine before the COVID-19 pandemic to avail neurological consultations, and all were able to conduct the telemedicine follow-up visit without requiring technical assistance, even in instances in which another person was present during the remote consultation (37.50%). Seven patients (87.50%) lived in Northern Italy, whereas only one patient lived in Central-Southern Italy. All patients received healthcare in Northern Italy. Seven patients (87.50%) reported being retired, whereas one patient (12.50%) was employed (Table 6).

**Table 6:** Demographic and clinical data of patients who independently completed the telephone survey

	Demographic and clinical data	8 Patients
Age (years)^a^		73.00 ± 8.52
Education (years)^a^		14.25 ± 2.31
Gender ^b^	Male	4 (50.00)
	Female	4 (50.00)
Occupation^b^	Working	1 (12.50)
	Retired	7 (87.50)
	Unemployed	0 (0.00)
MMSE score^a,c^		28.88 ± 0.83
Disease duration (years)^a^		3.86 ± 0.9
Behavioural disorders perceived by the caregiver ^b^	Slight	2 (25.00)
	Moderate	1 (12.50)
	Severe	0 (0.00)
	Absent	5 (62.50)
Area of residency^b^	Northern Italy	7 (87.50)
	Central-Southern Italy	1 (12.50)
Previous use of telemedicine^b^	Yes	0 (0.00)
	No	8 (100.00)
Telemedicine technical support^b^	Yes	0 (0.00)
	No	8 (100.00)
Neurological assistance at area of residency^b^	Yes	7 (87.50)
	No	1(12.50)
Area of neurological assistance^b^	Northern Italy	8 (100.00)
	Central-Southern Italy	0 (0.00)

^a^Data are expressed as mean ± standard deviation

^b^Data are expressed as frequency (%)

^c^MMSE: Mini Mental State Examination

According to the responses collected on the ‘*usefulness*/*perceived usefulness*’ construct investigated by the telephone survey, this sample of patients reported that the neurological telemedicine consultation was overall useful (87.50%, ‘strongly useful’ and 12.50%, ‘quite useful’). Regarding ‘*technical* *quality*’, 87.50% reported excellent technical quality during telemedicine visits and 12.50% reported good technical quality. With regard to the ‘*effectiveness*’, telemedicine consultations were mostly perceived as effective by patients (87.50%, ‘very effective’ and 12.50%, ‘quite effective’). When evaluating the ‘*effect on interaction*’, all patients found the interaction with the neurologist to be excellent. Moreover, on the ‘*experience*’ construct, patients reported that overall they had a positive experience (87.50%, ‘very good experience’ and 12.50%, ‘good experience’). Finally, regarding *‘satisfaction’*, all patients reported being completely satisfied of telemedicine consultation. The responses of patients did not differ statistically from those of caregivers (Table 4). No significant correlations were found between constructs examined.

## Discussion

This study evaluated the experience of telemedicine in patients with cognitive deficits and their caregivers. Although there was substantial heterogeneity in the participants’ characteristics, our results demonstrated that telemedicine for routine care was effective.

Due to the COVID-19 pandemic, public health measures taken to contain the spread of the virus created an urgent need to integrate virtual care into the existing healthcare infrastructure. In addition, the discontinuation of face-to-face activities, including rehabilitation sessions, medical appointments, and programmes, has had a detrimental effect on cognition and exacerbated stereotypic behaviours (e.g., strict adherence to routine) in this vulnerable population and increased the pressure on caregivers [2].

Our data showed that patients and caregivers were highly satisfied with the neurological video consultation, with an overall satisfaction rate of approximately 98.15% for caregivers and 100% for patients. This supports previous findings that telemedicine is a useful tool in dementia healthcare for supporting caregivers and reducing their burden [8]. According to Europe’s Alzheimer’s Society, telemedicine has also helped patients build a good support network, kept patients well informed, ensured that medical supplies were adequate, and enabled patients to remain physically and mentally active and stay socially connected [9]. In this context, a reduction in social contact and access to health services has encouraged the use of telemedicine to reduce the risk of developing negative mental health outcomes, improve dementia symptom management, mainly AD, and provide mental health care. Moreover, it can help caregivers by providing useful guidance on non-pharmacological measures to control symptoms affected by the new confinement measures. As the disease progresses, patients become more dependent on their caregivers and experience a loss of autonomy in their activities of daily life. The relatives of patients with chronic neurological conditions are assumed to be the primary caregivers in non-medical settings. Moreover, caregivers provide physical and emotional support to patients and play key roles in their decision-making processes [10]. In the management of chronic diseases, the well-being of caregivers is crucial, as a high level of burden on them may lead to a breakdown in care. Therefore, patients and caregivers must be educated on the management of cognitive impairment [11]. Additionally, telemedicine allows health support in real-time, even from a distance, making adequate medication adjustments possible when necessary without exposing patients and caregivers to the risk of COVID-19 infection.

The responses also highlighted that caregivers found video consultation ‘very useful’ in 87.04% of cases, while they found it ‘very effective’ in only 68.52% of cases. In agreement with the guidelines of Langbecker et al. [6], in our survey, for the construct ‘*usefulness/perceived usefulness*’, we evaluated how an interaction by telemedicine can produce benefits; for example, perceived convenience, time and cost saving, or the effect on the continuity of care. In contrast, for the construct ‘*effectiveness*’ we focused on assessing whether a video consultation interaction helped improve the health status of a patient; for example, patient well-being, change in health status, quality of life, or patient empowerment (see Table 1). Therefore, this difference can be explained by the different items measured for the two constructs. However, comparison studies evaluating face-to-face visits and video consultations are needed to understand whether the lowest score to the ‘*effectiveness*’ construct is due to the modality in which the medical visit was delivered, or by the progressive and irreversible nature of the dementia disease. Previous studies have demonstrated that telemedicine can be more clinically effective than usual methods of care [12]; however, the available evidence is discipline-specific, which highlights the need for more studies on the clinical effectiveness of video consultation across a wider spectrum of clinical health services. These findings support the view that in the right context, telemedicine will not compromise the effectiveness of clinical care compared to conventional forms of health service delivery [12].

Overall, the data suggest that the video consultation was very useful for the participants because it reduced the burden of transportation and trips outside the home for persons with dementia and their caregivers. This is consistent with previous studies conducted in urban and rural contexts [13, 14]. We found that video consultations were beneficial in terms of financial and travel time burdens, although our sample mainly comprised people living in a metropolitan area. Another reason for the lower total travel time expenses in our sample was that almost all caregivers were family members, who were either employed or who took time off work to take their relatives to medical appointments. Previous studies [15, 16] have shown that telemedicine consultations for monitoring chronic conditions are a convenient approach because patients can avoid travelling to medical facilities for routine follow-up appointments. They are more convenient and safer for patients, and less burdensome for caregivers. The convenience of telemedicine may help mitigate these immediate difficulties and diminish care-partner stress, in part, by reducing barriers to seeking care and eliminating travel to medical appointments and other in-person therapies. In addition, the benefits of telemedicine have been reported in dementia subtypes, including frontotemporal dementia, where telemedicine has demonstrated validity as a triage tool for increasing practice outreach and efficiency [12].

Finally, although technological barriers, including lack of equipment and the inability of older adults to independently manipulate technologies, could have hindered the use of telemedicine, our results showed that most participants, including older respondents, were able to access the platform. This proves that the pandemic has accelerated the digitisation process of care. Care partners can play a key role in optimising the home environment for telemedicine by minimising clutter and background noise, and simplifying tasks for patients with cognitive impairment. Orientation and training prior to an encounter can help set clear expectations for the care partner and reduce the severity of the situation for the person with cognitive impairment.

Despite telemedicine has demonstrated many benefits during the current crisis [7] owing to the pandemic, long-term considerations to address barriers to telemedicine care deserve equal attention because of its potential to improve the accessibility of care for patients with dementia and their caregivers.

## Limitations

The purpose of our study was to describe the experience of video consultations for patients with cognitive deficits and caregiver. However, the small sample size and the absence of a control group attending face-to-face consultations limited the generalisability of our data. A large multicentre study is required to further examine the application and effectiveness of this type of intervention.

Our sample population mainly inhabited urban areas; however, underserved populations (e.g. those from rural areas) may benefit more from this intervention.

Future research should aim to assess the feasibility of telemedicine for patients living in both remote and rural areas to address this limitation of the current study.

## Conclusions

Our results provide baseline information for designing improved telemedicine services that can open paths to achieving the goal of a user-friendly and satisfactory experience of virtual care delivery for patients with cognitive impairment and their caregivers. Telemedicine is well received by patients and care partners, but successful delivery requires support staff and care partners to navigate technologies. The benefits of telemedicine are several and promising, and it has several possible applications in the management of frail patients. We believe that the application of technology in medicine is useful for patients, healthcare providers, and institutions, allowing easy access to medical services, avoiding stressful travel, and reducing healthcare costs and waiting lists. Future studies should address this landscape to improve the quality of service, discover the best method of use, and expand its use among frail patients.

## Data Availability

The datasets generated and analysed during the current study are available from the corresponding author on reasonable request.
